# Research on the Trajectory Planning of Demolition Robot Attachment Changing

**DOI:** 10.3390/s20164502

**Published:** 2020-08-12

**Authors:** Qian Deng, Shuliang Zou, Hongbin Chen, Weixiong Duan

**Affiliations:** 1 College of Mechanical Engineering, University of South China, Hengyang 421001, China; dengqian@usc.edu.cn (Q.D.); chenhb2021@163.com (H.C.); duanwx2020@126.com (W.D.); 2 Hunan Provincial Key Laboratory of Emergency Safety Technology and Equipment for Nuclear Facilities, University of South China, Hengyang 421001, China

**Keywords:** demolition robot, attachment changing, trajectory planning, inverse kinematics

## Abstract

The process of changing the attachment of a demolition robot is a complex operation and requires a high docking accuracy, so it is hard for operators to control this process remotely through the camera’s perspective. To solve this problem, this paper studies trajectory planning for changing a demolition robot attachment. This paper establishes a link parameter model of the demolition robot; the position and attitude of the attachment are obtained through a camera, the optimal docking point is calculated to minimize the distance error during angle alignment for attachment change, the inverse kinemics of the demolition robot are solved, the trajectory planning algorithm and visualization program are programmed, and then the trajectory planning for the demolition robot attachment changing method is proposed. The results of calculations and experiments show that the method in this paper can meet the accuracy, efficiency, and safety requirements of demolition robot attachment changing, and it has promising application prospects in the decommissioning and dismantling of nuclear facilities and other radioactive environments.

## 1. Introduction

The first remote control hydraulic demolition robot designed for working in dangerous environments was developed in the 1970s and is widely applied for purposes such as nuclear accident emergency response and the decommissioning of nuclear facilities [1,2,3]. Compared with traditional construction machinery, the output torque of the demolition robot is larger and the operation ability is stronger. A demolition robot can enter and work in high-risk environments that are too dangerous for humans to enter, which broadens the application scope of the demolition robot and improves the dismantling operation efficiency [4,5,6,7]. In the field of demolition robots, BROKK from Sweden is one of the world’s leading manufacturers and has developed more than 15 types of demolition robots [8]. FINMAC from Finland developed the F16 demolition robot, a company in Japan developed the Tmsuk T52 and T53 dual arm robots, TOPTEC from Germany developed the TOPTEC1850E and TOPTEC2500E robots, and GIANT HYDRAULIC TECH from China developed the GTC15 and GTC30 robots [9].

In order to make the demolition robot more suitable for operation in nuclear environments, researchers have carried out a lot of work on demolition robots. A remote-control graphic transmission system was developed, and the operator can freely adjust the camera angle to observe the situation around the dismantled robot as needed [10]. A fault diagnosis and human–computer interaction system for a demolition robot was designed [11,12]. A demolition robot system for nuclear waste identification and capture was developed [13]. Another advantage of the demolition robots used in the nuclear industry is that they are multifunctional, and the attachments of a demolition robot can be changed according to different working conditions.

There are two types of methods for changing the attachment of demolition robots, the first of which is manual attachment changing, and the other is remote attachment changing. In the manual attachment changing type of demolition robot, the connection mode and structure of the robot and attachment are similar to those of construction machinery, such as an excavator. In the process of attachment changing, it is necessary to manually complete the assembly of the mechanical structure and hydraulic oil circuit of the attachment and robot. This type of robot is not suitable for working in a radioactive environment because of its radioactive contamination.

**Figure 1:**
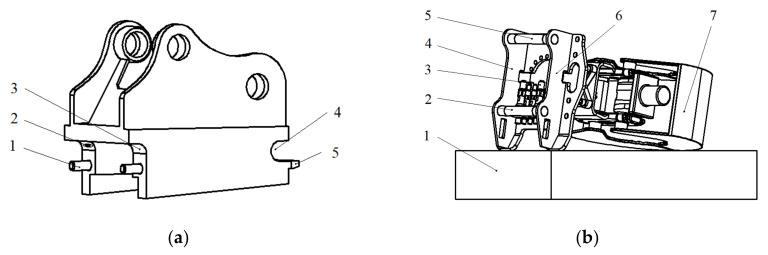
The quick-hitch equipment of a demolition robot and attachment. (**a**) Quick-hitch equipment: (1) lock pin shaft, (2) contact switch, (3) fixed side of lower shaft, (4) fixed side of upper shaft, and (5) guide plate. (**b**) Attachment: (1) carriage, (2) lower shaft, (3) hydraulic quick connector (male), (4) left side plate, (5) upper shaft, (6) right side plate, and (7) grabber.

In the remote attachment changing type of demolition robot, the operator does not need to touch the robot or the attachment during the process of attachment changing. The quick-hitch equipment of a demolition robot and the attachment structure are shown in Figure 1. When the fixed side of the upper shaft of the quick-hitch is docking with the upper shaft of the attachment, and the fixed side of the lower shaft of the quick-hitch is docking with the lower shaft of attachment, the lock pin shaft locks the lower shaft and drives the coupling of the quick-hitch equipment hydraulic quick connector (female) with the attachment hydraulic quick connector (male).

**Figure 2:**
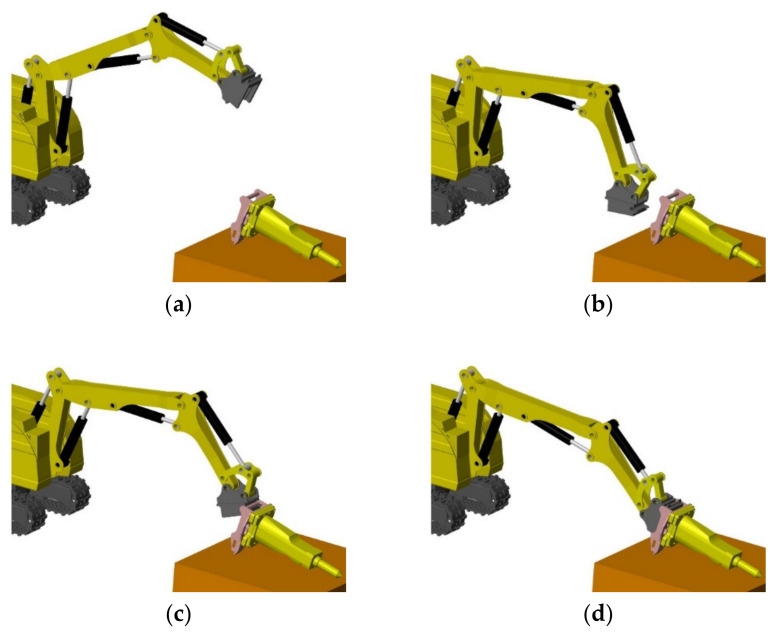
Attachment changing procedure: (**a**) initialization, (**b**) preparation, (**c**) range alignment, and (**d**) angle alignment.

There are four procedures for remotely changing the attachment of a demolition robot: initialization, preparation, range alignment, and angle alignment. In the initialization stage, as shown in Figure 2a, the robot moves to the changing area, and the quick-hitch equipment of the robot begins to dock with the attachment. In the preparation stage, as shown in Figure 2b, the quick-hitch equipment moves closer to the attachment, and the pose of the quick-hitch equipment should be adjusted. In the range alignment stage, as shown in Figure 2c, the quick-hitch equipment of the robot moves to the attachment docking spot, and the coordinate origin of the quick-hitch equipment overlaps with the attachment’s coordinate origin. In the angle alignment stage, as shown in Figure 2d, the quick-hitch equipment is manipulated to assemble the quick-hitch equipment and the attachment. When this stage is finished, the quick-hitch equipment and the attachment lock up and hydraulic circuit are enabled, thereby completing the attachment change. During the angle alignment stage, the complex movement of the robot’s quick-hitch equipment is achieved by the movement of two cylinders. Therefore, the process of changing the attachment of a demolition robot is a complex operation and requires a high docking accuracy, and it depends on the level and experience of the operator. For the reasons discussed above, there is widespread concern regarding the structure, reliability, and other practical engineering problems of the attachment and quick-hitch equipment; the attachment changing process for both types of demolition robots is characterized by low automation and intelligence.

To solve the issues above, the forward kinematics and inverse kinematics of the demolition robot need to be established and solved [14,15,16,17,18]. The position and attitude of the attachment also need to be obtained and can be achieved by using visual servo technology [19,20,21,22]. In our previous work, an error compensation method for changing the attachment of demolition robots was proposed, and a visualization system for the remote changing of the demolition robot attachment was developed [23,24]. After the coordinate systems of the robot’s quick-hitch equipment and the attachment are obtained accurately, the motion trajectory of the quick-hitch equipment should be analyzed and planned [25] so as to provide the optimal attachment changing solution to the operator. In recent works on robot path planning research, a method for constrained motion planning based on vision was proposed, which enables a robot to move its end-effector over an observed surface [26]. A new neural network model that uses a super twisting algorithm for the tracking control of mobile robot manipulators was proposed [27]. A method for effectively planning the motion trajectory of robots in manufacturing tasks was proposed, the tool-paths of which are usually complex and have a large number of discrete-time constraints as waypoints [28]. This study aimed to propose a method for remotely changing a demolition robot’s attachment. The proposed method can reduce the complexity of the attachment changing process for existing demolition robots and improve the degree of automatization of demolition robot attachment changing. The main contributions of this paper are illustrated in the following points:

- The range of the relative distance between the robot base coordinate frame {*B*} and the attachment coordinate frame {*T*} is given, and the optimal distance interval is proposed.
- The optimal position of joint {4} is calculated, and the joint angles of the robot for attachment changing are solved through inverse kinematics.
- A method for changing the demolition robot attachment by single joint motion is proposed, and the distance error of trajectory between {*W*} and {*T*} is minimized.

In this paper, Section 1 introduces the principle and difficulties of changing a demolition attachment. In Section 2, the demolition robot model is described, and the forward kinematics equation is solved. In Section 3, the motion trajectory of changing the demolition robot attachment is studied, and the remote control of the attachment changing process with a trajectory planning method is proposed. In Section 4, comparative experiments of the trajectory planning method are reported. Section 5 provides a summary and describes future works.

## 2. Forward Kinematics of the Demolition Robot

**Figure 3:**
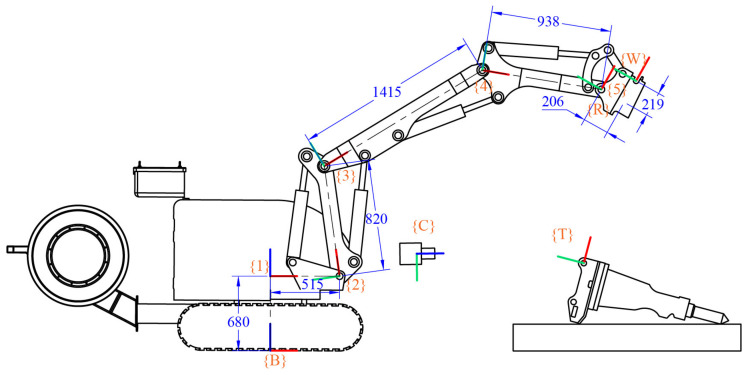
Demolition robot model. {*B*} is the robot’s base coordinate frame, {*W*} is the quick-hitch docking spot coordinate frame, {*C*} is the camera coordinate frame, {*T*} is the attachment docking spot coordinate frame, and {*R*} is the error compensation reference coordinate frame (the red axis is the X-axis, the green axis is the Y-axis, and the blue axis is the Z-axis).

The Denavit–Hartenberg (D-H) parameters should be described before the kinematic analysis of the demolition robot. The whole link of the demolition robot arm is connected by a set of connecting rods through the joints, and the five joints are all revolute joints. By establishing the modified D-H parameters of the demolition robot [29,30], the relative angles and positions of the links can be solved. The demolition robot’s model is shown in Figure 3, and the D-H parameters are shown in Table 1. The base coordinate frame {*B*} is set at the bottom-center of the robot tracked mobile platform; the X-axis of {*B*} is the forward direction, and the Z-axis of {*B*} is upwards. Joint {1} is the robot chassis rotatory joint, and the Z-axis of {1} is upwards and overlaps with the axis of the robot chassis rotatory joint, and the X-axis of {1} is parallel to the X-axis of {*B*}. Joint {2} is the upper arm rotatory joint driven by the upper arm cylinder; the X-axis direction of {2} is from joint {2} to joint {3} and overlaps with the connecting line between joint {2} and joint {3}, and the Z-axis of {2} overlaps with the axis of the upper arm rotatory joint and it is vertical paper inward. Joint {3} is the middle arm rotatory joint driven by the middle arm cylinder; the X-axis direction of {3} is from joint {3} to joint {4} and overlaps with the connecting line between joint {3} and joint {4}. The Z-axis of {3} overlaps with the axis of the middle arm rotatory joint. Joint {4} is the fore arm rotatory joint driven by the fore arm cylinder; the X-axis direction of {4} is from joint {4} to joint {5}, and it overlaps with the connecting line between joint {4} and joint {5}. The Z-axis of {4} overlaps with the axis of the fore arm rotatory joint. Joint {5} is the quick-hitch equipment rotatory joint driven by the quick-hitch equipment cylinder; the X-axis direction of {5} is parallel to the X-axis direction of {*W*}, and the Z-axis of {5} overlaps with the axis of the quick-hitch equipment rotatory joint. {*W*} is the quick-hitch docking spot coordinate frame, and the axis direction is determined by the structure of the quick-hitch equipment, as shown in Figure 3. {*T*} is the attachment docking spot coordinate frame when the attachment is connected to the quick-hitch equipment, and {*W*} overlaps with {*T*}.

*θ*_1_, *θ*_2_, *θ*_3_, *θ*_4_, and *θ*_5_ are the rotation angles of joints {1}, {2}, {3}, {4}, and {5}, respectively. The lengths of the links between the joints are $l_{1}$ = 0.680 m, $l_{2}$ = 0.515 m, $l_{3}$ = 0.820 m, $l_{4}$ = 1.415 m, $l_{5}$ = 0.938 m, $W_{x}$ = 0.219 m, and $W_{y}$ = 0.206 m. T4B denotes the homogeneous transformation matrix from the base coordinate frame {*B*} to the joint {4} coordinate frame, which is shown in Equation (1). TWB denotes the homogeneous transformation matrix from the base coordinate frame {*B*} to the robot quick-hitch equipment docking coordinate frame {*W*}, which is shown in Equation (2).

T1B=cosθ1−sinθ100sinθ1cosθ100001l10001,T21=cosθ2−sinθ20l200−10sinθ2cosθ2000001,

T32=cosθ3−sinθ30l3sinθ3cosθ30000100001,T43=cosθ4−sinθ40l4sinθ4cosθ40000100001,

T54=cosθ5−sinθ50l5sinθ5cosθ50000100001,T5W=100wx010−wy00100001.

T4B=T1BT21T32T43

(1) $$=\left[\begin{array}{cccc} c_{234}c_{1} & -s_{234}c_{1} & s_{1} & \left(l_{2}+l_{3}c_{2}+l_{4}c_{23}\right)c_{1}\\ c_{234}s_{1} & -s_{234}s_{1} & -c_{1} & \left(l_{2}+l_{3}c_{2}+l_{4}c_{23}\right)s_{1}\\ s_{234} & c_{234} & 0 & l_{1}+l_{3}s_{2}+l_{4}s_{23}\\ 0 & 0 & 0 & 1 \end{array}\right],$$

TWB=T1BT21T32T43T54TW5 

(2) $$=\left[\begin{array}{cccc} c_{2345}c_{1} & -s_{2345}c_{1} & \;s_{1} & \;\left(l_{2}+l_{3}c_{2}+l_{4}c_{23}+l_{5}c_{234}\right)c_{1}+w_{x}c_{2345}c_{1}+w_{y}s_{2345}c_{1}\\ c_{2345}s_{1} & -s_{2345}s_{1} & -c_{1} & \;\left(l_{2}+l_{3}c_{2}+l_{4}c_{23}+l_{5}c_{234}\right)s_{1}+w_{x}c_{2345}s_{1}+w_{y}s_{2345}s_{1}\\ s_{2345} & \;c_{2345} & \;0 & \;l_{1}+l_{3}s_{2}+l_{4}s_{23}+l_{5}s_{234}+w_{x}s_{2345}-w_{y}c_{2345}\\ 0 & 0 & 0 & 1 \end{array}\right]$$

where:

$$s_{1}=sin\theta_{1},c_{1}=cos\theta_{1},s_{23}=\sin\left(\theta_{2}+\theta_{3}\right),c_{2345}=\cos\left(\theta_{2}+\theta_{3}+\theta_{4}+\theta_{5}\right),\cdots$$

$$l_{1}=0.68\;m,l_{2}=0.515\;m,l_{3}=0.82\;m,l_{4}=1.415\;m,$$

$$l_{5}=0.938\;m,w_{x}=0.219\;m,w_{y}=0.206\;m.$$

{*R*} is the reference coordinate frame that is installed on the quick-hitch equipment. The purpose of introducing the reference coordinate frame {*R*} is to compensate for the measurement error. TTW denotes the homogeneous transformation matrix from the quick-hitch equipment docking coordinate frame {*W*} to the attachment docking spot coordinate frame {*T*}, which is shown in Equation (3).

(3) TTW=TRWTTR=TRWTRC−1TTC.

In Equation (3), TRW is a constant term, and TRC and TTC are the coordinate data collected by the camera.

## 3. Trajectory Planning of Attachment Changing

### 3.1. Position Determination of Joint {4}

**Figure 4:**
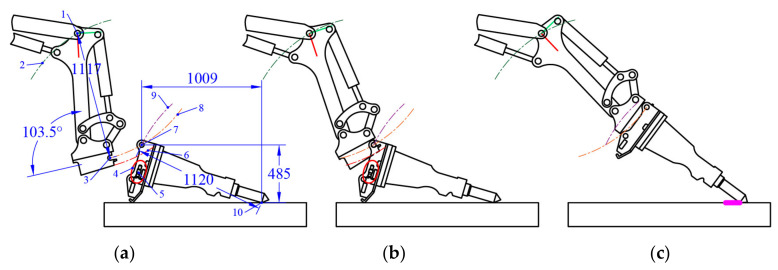
Trajectory of attachment changing. (**a**) Preparation: (1) joint {4}, (2) trajectory of joint {4}, (3) {*W*}, (4) collision region, (5) hydraulic quick coupling (male) of the attachment, (6) trajectory of quick hitch equipment edge, (7) {*T*}, (8) trajectory of {*W*}, (9) trajectory of {*T*}, (10) attachment support point, (**b**) range alignment, and (**c**) angle alignment.

In Figure 4a, the {*T*} position is the center of the arc drawn with the green dot-dash line, and the distance between {4} and {*W*} is the radius. If the joint {4} position is on this arc, {*W*} will perform an arc movement around {4} by rotating joint {4}; the motion trajectory of {*W*} is shown in Figure 4a with orange dot-dash line. If the {*T*} position is on the motion trajectory of {*W*}, range alignment for the attachment changing can be completed, as shown in Figure 4b. In order to ensure that the quick hitch equipment does not collide with the hydraulic quick coupling (male) of the attachment, the trajectory of the quick-hitch equipment edge must not enter the collision region. When the docking is completed, joint {4} continues to rotate counter-clockwise, the attachment is lifted by the quick hitch equipment, and {*T*} rotates clockwise around the supporting point of attachment, as shown in Figure 4b with the purple dot-dash line. The motion trajectories of {*W*} and {*T*} do not overlap. However, due to the structural constraints of the quick-hitch equipment, the attachment is forced to move in the horizontal direction during the angle alignment, and {*W*} and {*T*} will only rotate relative to each other. Figure 4c shows the completion of angle alignment, and the magenta line segment is the horizontal movement distance of the attachment support point.

When the rotation angle of joint {5} is 103.5°, the X-axis of {*W*} is tangent to the trajectory of {*W*}, the distance between {4} and {*W*} is 1.117 m, and the distance between {4} and the quick-hitch equipment edge is 1.185 m. Assuming that the coordinates of {*T*} are ($X_{T}$, 0, $Z_{T}$) and $R_{z}$ is the rotation angle around its Z-axis, the trajectory of joint {4} is:

(4) $$\left\{\begin{array}{c} x_{4}=1.117cos\theta_{4}+X_{T}\\ z_{4}=1.117sin\theta_{4}+Z_{T} \end{array}\right..$$

{*T*} spans −0.223 m along the X-axis and −0.04 m along the Z-axis, which is the elliptical center of the collision region. The elliptic equation is:

(5) $$\left\{\begin{array}{c} x_{e}=0.096cos\theta_{e}cosRZ_{T}-0.06sin\theta_{e}sinRZ_{T}+X_{T}-0.223cosRZ_{T}+0.04sinRZ_{T}\\ z_{e}=0.096cos\theta_{e}sinRZ_{T}-0.06sin\theta_{e}cosRZ_{T}+Z_{T}-0.223sinRZ_{T}-0.04cosRZ_{T} \end{array}\right..$$

Assuming that the coordinates of {4} are ($X_{4}$, 0, $Z_{4}$), the trajectory of {*W*} is:

(6) $${\left(x_{W}-X_{4}\right)}^{2}+{\left(z_{W}-Z_{4}\right)}^{2}=1.117^{2}.$$

If $z_{W}$ is known, then:

(7) $$x_{W}=\sqrt{1.117^{2}-{\left(z_{W}-Z_{4}\right)}^{2}}+X_{4}.$$

The trajectory of the quick-hitch equipment edge is:

(8) $$\left\{\begin{array}{c} x_{b}=1.185cos\theta_{b}+X_{4}\\ z_{b}=1.185sin\theta_{b}+Z_{4} \end{array}\right..$$

The radius of rotation around the crushing hammer support point $A_{R}$ is 1.120 m, and the Z-direction distance between {*T*} and the crushing hammer support point $A_{Z}$ is 0.485 m, as shown in Figure 4a. The trajectory of {*T*} is:

(9) $$\left\{\begin{array}{c} x_{T}=A_{R}\cdot cos\theta_{T}+X_{T}+A_{X}\\ z_{T}=A_{R}\cdot sin\theta_{T}+Z_{T}+A_{Z} \end{array}\right..$$

An algorithm program was compiled to calculate the coordinate position of {4}. The specific algorithm is shown in Algorithm 1. In Algorithm 1, a constant term is introduced in the *RZ*_4_ solution process, which reserves a certain rotation angle for the range alignment stage.
**Algorithm 1** Calculate the optimal joint position of joint {4}.**Inputs:** Position and attitude of {*T*}, $X_{T}$, $Z_{T}$, $RZ_{T}$. Data of attachment, $A_{X}$, $A_{Z}$, $A_{R}$.**Outputs:** Position and attitude of {4}, $X_{4}$, $Z_{4}$, $RZ_{4}$.**Notes:**$\Delta \theta_{T}=\left\{0,\cdots ,-\frac{\pi }{6}\right\}$, $\theta_{e}=\left\{0,\cdots ,2\pi \right\}$, $\theta_{4}=\left\{\underset{N}{\underset{⏟}{\frac{\pi }{2},\cdots ,\pi }}\right\}$, $\Delta \theta_{b}=\left\{-\frac{\pi }{2},\cdots ,0\right\}$.1.$\theta_{T}=\pi -arctan2\left(A_{Z},A_{X}\right)+\Delta \theta_{T}$.2.Calculate the trajectory of {*T*} according to Equation (9), and get $x_{T}$, $z_{T}$.3.Calculate the collision region ellipse according to Equation (8), and get $x_{e}$, $z_{e}$.4.Calculate the trajectory of {4} according to Equation (7), and get
$x_{4}$, $z_{4}$.5.**for**i=1,⋯,N6.$\;X_{4}=x_{4}^{i}$, $Z_{4}=z_{4}^{i}$7$\;z_{W}=z_{T}$, calculate $x_{W}$, according to Equation (4)8.$\;\theta_{b}=-\arctan2\left(Z_{4}-Z_{T},X_{4}-X_{T}\right)+\Delta \theta_{b}$9. Calculate the trajectory of the quick-hitch equipment edge according to Equation (6), and get
$x_{b}$, $z_{b}$.10.$\;d^{i}=max\left(\sqrt{{\left(x_{T}-x_{W}\right)}^{2}+{\left(z_{T}-z_{W}\right)}^{2}}\right)$.11. **if** arc ($x_{b},\;z_{b}$) is tangent to ellipse ($x_{e},\;z_{e}$), **then**12.
  
**break**
13. **end if**14.**end for**15.$\left[index,\;d^{min}\right]=min\left(d\right)$16.$X_{4}=x_{4}^{index}$, $Z_{4}=z_{4}^{index}$17.$RZ_{4}=arctan2\left(Z_{T}-Z_{4},X_{T}-X_{4}\right)-0.236-\frac{\pi }{10}$18.**Output**$X_{4}$, $Z_{4}$, $RZ_{4}$.

**Figure 5:**
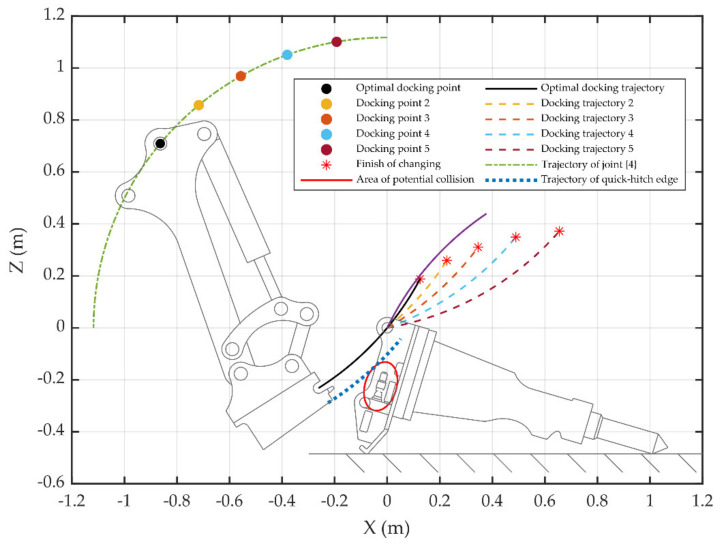
Position determination of joint {4}.

If the breaking hammer is placed horizontally, the position of {*T*} is (0, 0, 0), and the rotation angle around its Z-axis is 73.5°, as shown in Figure 5. According to Algorithm 1, the optimal docking point of {4} is (−0.864 m, 0, 0.709 m), RZ_4_ is −70.9°, and the maximum trajectory distance error between {*W*} and {*T*} is 0.017 m. Table 1 shows the data of other docking points. In Table 2, the distance error difference between docking point 1, the optimal docking point, and docking point 5 is 0.36 m. Joint {4} at docking point 5 needs to rotate with the largest angle during the angle alignment stage, and the attachment is in danger of tipping. After calculating the optimal docking point, the next step is to study how to move joint {4} to this point.

### 3.2. Inverse Kinemics of Demolition Robot Attachment Changing

If the breaking hammer is placed horizontally, the position of {*T*} is (2.8 m, 0, 0.485 m), and the rotation angle around its Z-axis is 73.5°. According to Algorithm 1, the optimal docking point of {4} is (1.936 m, 0, 1.195 m), and *RZ*_4_ is −70.9°. Putting $\theta_{1}=0$ into Equation (1),

(10) T4B=c234−s2340l2+l3c2+l4c2300−10s234c2340l1+l3s2+l4s230001,

(11) $$X_{4}=l_{2}+l_{3}c_{2}+l_{4}c_{23},$$

(12) $$Z_{4}=l_{1}+l_{3}s_{2}+l_{4}s_{23}.$$

The constant terms in Equations (11) and (12) are shifted to the left, and then squared and added to get:

$${\left(X_{4}-l_{2}\right)}^{2}+{\left(Z_{4}-l_{1}\right)}^{2}=l_{3}^{2}+l_{4}^{2}+2l_{3}l_{4}\left(c_{2}c_{23}+s_{2}s_{23}\right)$$

(13) $$=l_{3}^{2}+l_{4}^{2}+2l_{3}l_{4}\left(c_{2}^{2}c_{3}-s_{2}c_{2}s_{3}+s_{2}c_{2}s_{3}+s_{2}^{2}c_{3}\right)=l_{3}^{2}+l_{4}^{2}+2l_{3}l_{4}c_{3},$$

(14) $$c_{3}=\frac{{\left(X_{4}-l_{2}\right)}^{2}+{\left(Z_{4}-l_{1}\right)}^{2}-l_{3}^{2}-l_{4}^{2}}{2l_{3}l_{4}},$$

(15) $$\theta_{3}=arctan2\left(c_{3},\pm \sqrt{1-c_{3}^{2}}\right).$$

Putting the constant terms into Equations (13) and (14) gives $\theta_{3}=\pm 99.6{}^{\circ}$, because of $\theta_{3}\leq 20{}^{\circ}$, $\theta_{3}=-99.6{}^{\circ}$. By expanding Equations (11) and (12), the following is obtained:

(16) $$X_{4}-l_{2}=l_{3}c_{2}+l_{4}c_{2}c_{3}-l_{4}s_{2}s_{3}=\left(l_{3}+l_{4}c_{3}\right)c_{2}-l_{4}s_{3}s_{2},$$

(17) $$Z_{4}-l_{1}=l_{3}s_{2}+l_{4}c_{2}s_{3}+l_{4}s_{2}c_{3}=\left(l_{3}+l_{4}c_{3}\right)s_{2}+l_{4}s_{3}c_{2},$$

where:

(18) $$rcos\gamma =l_{3}+l_{4}c_{3},$$

(19) $$rsin\gamma =l_{4}s_{3}.$$

Putting Equations (18) and (19) into Equations (16) and (17) gives:

(20) $$\frac{X_{4}-l_{2}}{r}=cos\gamma cos\theta_{2}-sin\gamma sin\theta_{2}=cos\left(\gamma +\theta_{2}\right),$$

(21) $$\frac{Z_{4}-l_{1}}{r}=cos\gamma sin\theta_{2}+sin\gamma cos\theta_{2}=sin\left(\gamma +\theta_{2}\right),$$

(22) $$\gamma +\theta_{2}=arctan2\left(\frac{Z_{4}-l_{1}}{r},\frac{X_{4}-l_{2}}{r}\right),$$

(23) $$\theta_{2}=arctan2\left(Z_{4}-l_{1},X_{4}-l_{2}\right)-arctan2\left(l_{4}s_{3},l_{3}+l_{4}c_{3}\right).$$

Putting the constant terms in Equation (23) gives $\theta_{2}=87.3{}^{\circ}$, because:

(24) $$RZ_{4}=\theta_{2}+\theta_{3}+\theta_{4}.$$

**Figure 6:**
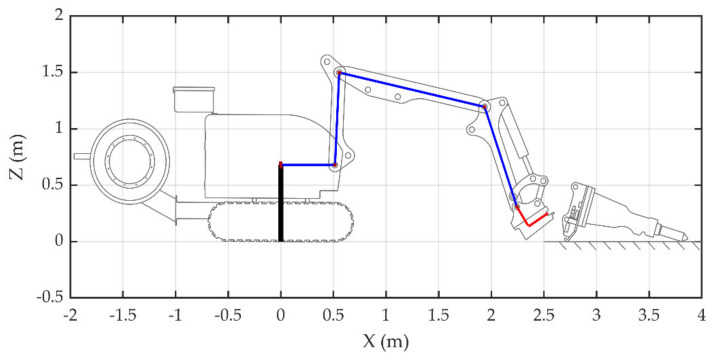
Preparation of the demolition robot attachment changing process.

Therefore, $\theta_{4}=-58.6{}^{\circ}$. In Figure 6, the demolition robot joint {1} angle $\theta_{1}$ is 0, the joint {2} angle $\theta_{2}$ is 87.3°, the joint {3} angle $\theta_{3}$ is −99.6°, the joint {4} angle $\theta_{4}$ is −58.6°, and the joint {5} angle $\theta_{5}$ is 103.5°. Assistance marker points are designed in the visualization interface to help the operator quickly control the joints to move to the specified positions. In this state, the optimal angle alignment described in Section 3.1 can be completed by rotating joint {4} counter-clockwise. When joint {4} reaches the specified position, the preparation stage of changing the attachment is completed.

### 3.3. Position Determination of the Attachment Docking Coordinate Frame {T}

In the process of attachment changing, the Z-axis of the quick-hitch equipment of {*W*} is parallel to the Z-axis of {*T*}. By moving the demolition robot, the Y-axis of {*B*} becomes parallel to the Z-axis of {*T*}. The distance between {*B*} and {*T*} also needs to be restricted. According to Equations (11) and (12), when the joint {4} position obtained by algorithm 1 satisfies Equation (24), all the joints of the robot can be manipulated to the specified position.

(25) $${\left(X_{4}-l_{2}\right)}^{2}+{\left(Z_{4}-l_{1}\right)}^{2}\leq l_{3}^{2}+l_{4}^{2}.$$

Since TTB satisfying the condition of the attachment changing process is not unique, the farther the distance between {*B*} and {*T*}, the farther the distance between the camera coordinate frame {*C*} and {*T*}, so the accuracy of obtaining the {*T*} position decreases. On the other hand, the demolition robot joints {2} to {4} have a rotation angle range, and it is necessary to restrict TTB to ensure a smooth attachment changing process.

**Figure 7:**
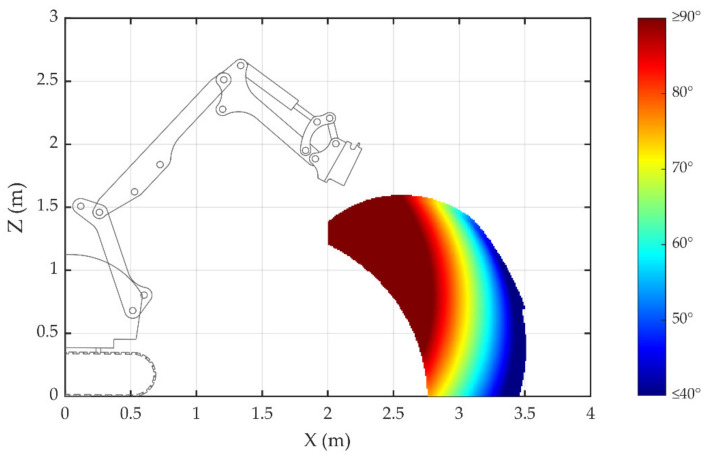
Position determination of attachment docking coordinate frame {*T*}.

If the breaking hammer is placed horizontally, the range of the {*T*} position along the X-axis is from 2 to 4 m, the range of the {*T*} position along the Z-axis is from 0 to 2 m, and the rotation angle around its Z-axis is 73.5°. The range of the rotation angle of joint {2} is from 30° to 140°, the range of joint {3} is from −108° to −18°, and the range of joint {4} is from −100° to 23°. The joint angle required for the distance between {*B*} and {*T*} is calculated according to the method introduced in Section 3.2 and Section 3.3, and the result is shown in Figure 7. Figure 7 takes {*B*} as the origin, and the attachment changing process can be completed in the colored area. The color in the colored area represents the angle of joint {2} for the attachment change: the dark red area indicates that the joint {2} angle is ≥90°, the red area indicates 80°, and the dark blue indicates ≤40°. In order to ensure that the camera obtains high-precision attitude information about {*T*}, the docking position of joint {4} should be in the deep red and red areas. For example, if the Z-axis coordinate of {*T*} is 0.485 m, the demolition robot should move to make the X-axis coordinate of {*T*} be between 2.66 m and 2.91 m. If the Z-axis coordinate of {*T*} is 1 m, the demolition robot should move to make the X-axis coordinate of {*T*} be between 2.3 m and 2.93 m.

An assistance wireframe is designed in the visualization interface to help the operator quickly move the robot to the specified position. When the robot arrives at the specified position, the initialization stage of changing the attachment is completed.

### 3.4. Process of Attachment Changing Trajectory Planning

**Figure 8:**
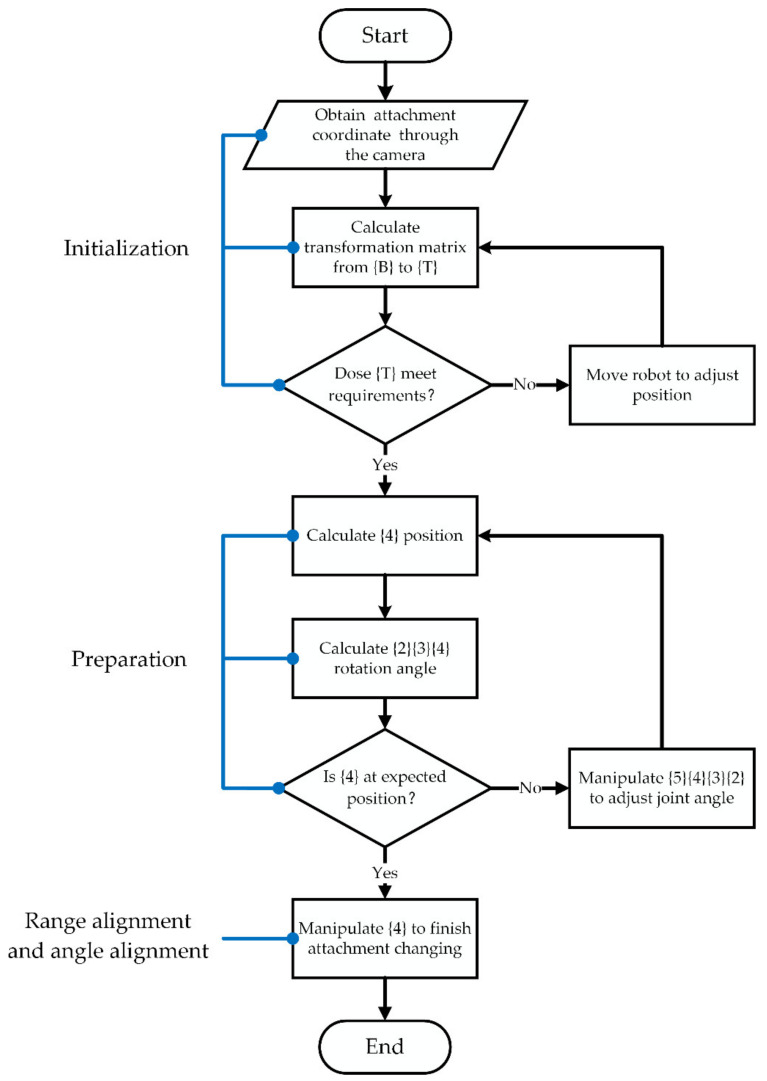
Block diagram of attachment changing trajectory planning.

The process of attachment changing trajectory planning is shown in Figure 8. In the initialization stage, the main task is to move the demolition robot to a suitable position for attachment changing. In the preparation stage, the joint {4} position and the joints {2} {3} {4} rotation angles are calculated, and the quick-hitch equipment coordinate frame {*W*} follows the optimal docking trajectory. Joint {4} is manipulated to rotate counterclockwise, and the range alignment and angle alignment stages are completed. The process of changing the demolition robot attachment is finished.

## 4. Experiment

### 4.1. Experimental Conditions

**Figure 9:**
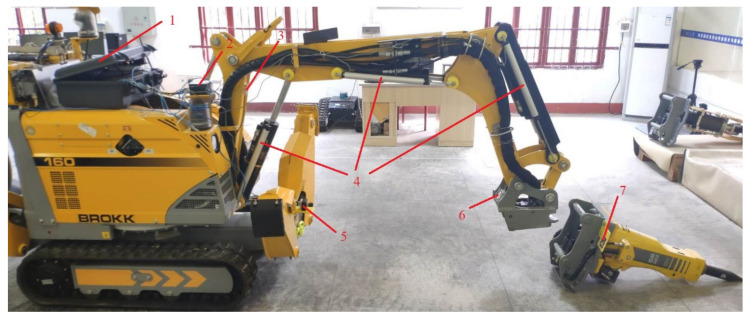
Attachment changing experimental conditions. (1) Nividia Jestson TX2 and sensors serial communication module, (2) wireless router, (3) inclinometer, (4) displacement sensors, (5) industrial camera, (6) error compensation reference tag, (7) positioning tag of attachment.

In this study, a trajectory planning toolkit for changing the demolition robot attachment was developed using the ros platform, which includes a robot visualization program, a robot cylinder length data acquisition and joint angle conversion program, a real-time error compensation program, and the trajectory planning program described in Section 3. In the experiments, the joint angle of the demolition robot was obtained by the sensors, and the attitude and position of the attachment were obtained by an industrial camera. The experiments involved remotely controlling the demolition robot to complete the attachment changing process indoors, and the attachment was placed on flat ground, which is shown in Figure 9.

### 4.2. Experimental Scene 1: Attachment Changing without Trajectory Planning

**Figure 10:**
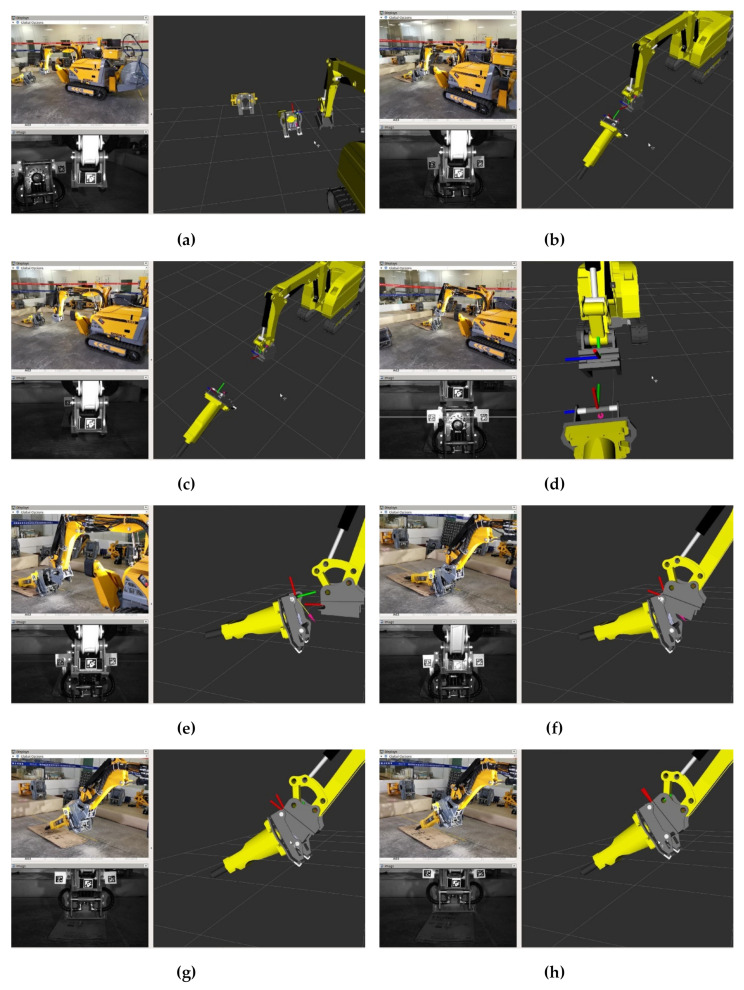
Experiment of attachment changing without trajectory planning. (**a**) (**b**) (**c**) and (**d**) Initialization stage. (**e**) Preparation stage. (**f**) Range alignment stage. (**g**) and (**h**) Angle alignment stage.

In Figure 10, the demolition robot, camera screen, and visualization interface are displayed together. The initiation stage of the attachment changing process is shown in Figure 10a–d. It was difficult to move the demolition robot to the docking position, and the operator controlled the robot only through the camera and visualization interface. The robot was in the wrong docking position, as shown in Figure 10b, and it went back and tried it once again, as shown in Figure 10c. When the Z-axis of the quick-hitch equipment of {*W*} was parallel to the Z-axis of {*T*}, it was in an appropriate docking position for the attachment changing, as shown in Figure 10d, and it needed to be verified by the operator through observation using the camera screen and visualization interface. Figure 10e shows the completion of the preparation stage; next, the range alignment stage was carried out. In the range alignment stage, the {*W*} position should be adjusted all the time to ensure that the quick-hitch equipment docks with the attachment. Figure 10f shows the completion of the range alignment. Figure 10g shows the angle alignment stage being carried out. Because the motion trajectories of {*W*} and {*T*} did not overlap, joints {4} and {5} were manipulated at the same time to ensure that the quick-hitch equipment and attachment were assembled smoothly. Figure 10h shows the completion of the attachment changing process. A video of the whole experiment can be found in Supplementary Materials.

### 4.3. Experimental Scene 2: Attachment Changing with Trajectory Planning

**Figure 11:**
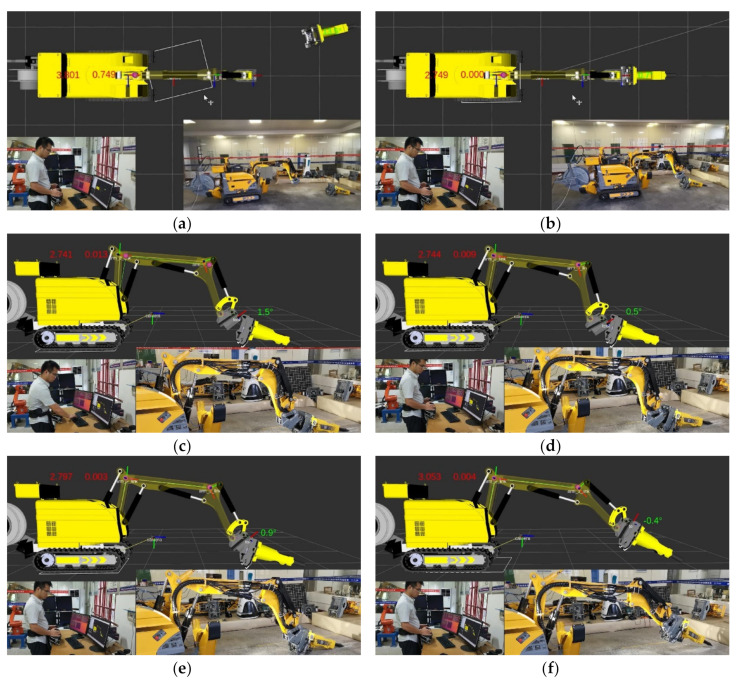
Experiment of attachment changing with trajectory planning. (**a**) Position determination of {*T*}. (**b**) Completion of initialization. (**c**) Trajectory planning. (**d**) The optimal docking position. (**e**) Completion of range alignment. (**f**) Completion of angle alignment.

In Figure 11, the operator, demolition robot, and visualization interface are displayed together. The robot was moved to the white wireframe position, which was calculated by the method described in Section 3.3, as shown in Figure 11a. In this way, the robot could move quickly to the designated position that satisfied the optimal configuration for the attachment change. When the robot arrived at the white wireframe, the initialization stage of the attachment changing process was completed, as shown in Figure 11b. In the preparation stage of the attachment changing process, the joint {4} position and joints {2} {3} {4} rotation angles were calculated by the method described in Section 3.1 and Section 3.2 For ease of remote control, the positions of joints {3} {4} were represented by purple spheres, as shown in Figure 11c. When the joints {3} {4} overlapped with the purple spheres, joint {4} was in the optimal docking position, and the preparation stage of the attachment changing process was completed, as shown in Figure 11d. In the range alignment and angle alignment stages, only joint {4} was manipulated quickly to finish the attachment changing process, and the distance error of the trajectory between {*W*} and {*T*} was at its minimum, as shown in Figure 11e,f. A video (Research on Trajectory Planning of Demolition Robot Attachment Changing) of the whole experiment is available online at https://youtu.be/4m-wow-ABio.

### 4.4. Discussion

Table 2 shows the time consumption of the attachment changing process with trajectory planning compared with the attachment changing process without trajectory planning. The time consumption can be reduced by 46% using the trajectory planning method in the attachment changing process. Experimental scene 1 adopted the error compensation algorithm, which could obtain high-precision data of TTW and provided the visualization interface for the operator to finish changing the attachment by remote control. However, during the initialization stage, the operator needed to move the demolition robot repeatedly to arrive at the appropriate docking position because the movement was based on qualitative observation. On the basis of experimental scene 1, experimental scene 2 added the trajectory planning algorithm, which could calculate the optimal docking position and provide quantitative assistance to complement the visualization interface. As shown in Table 3, during the initialization stage, experimental scene 2 saved 90 s compared with experimental scene 1. In the preparation stage, the time consumed by experiment 2 was not significantly different from that of experiment 1; the extra 10 s was mainly to adjust the joints {3} and {4} to precisely reach the set positions. In the range alignment and angle alignment stages, the advantage of experimental scene 2 over experimental scene 1 was that experiment 2 only needed to manipulate joint 4 to complete the remaining movement, avoiding the complex multi-joint composite movement in experimental scene 1. When the attachment was placed at different slopes or heights, the experimental scene 1 scheme may require many attempts and exercises to complete the attachment changing process, while the experiment scene 2 scheme only need the prompts from the visualization interface to complete the attachment changing process.

## 5. Conclusions

In this paper, the motion trajectory of changing a nuclear demolition robot attachment is studied. By calculating the optimal docking position of joint {4}, the inverse kinemics of the demolition robot were used to solve the coordinates of each joint, and the position of the robot base frame was determined. The proposed method for changing an attachment by remote control with a trajectory planning method was investigated through experiments. Compared with the existing attachment changing method, this proposed method did not need to manipulate multiple joints at the same time to complete complex motion, which reduced the operational difficulty of the range alignment and angle alignment in the process of attachment changing. On the other hand, the optimal docking of the attachment change was achieved, which minimized the distance error of the trajectory between the quick-hitch equipment and attachment during angle alignment, and it also ensured that no collision occurred between these two parts. The experimental results show that, at the same operating level, the time consumption in the process of changing the demolition robot attachment could be reduced by 46% by using the trajectory planning method. The method proposed in this paper improved the efficiency and safety of remotely changing a demolition robot attachment.

Since commercial demolition robots do not provide remote communication protocols, these can only be controlled by manual remote operation. Our group is developing an intelligent demolition robot called the Huluwa demolition robot. The Huluwa demolition robot, with high radiation resistance, will be equipped with a hydraulic servo control system, which has higher precision than an electro-hydraulic proportional control system. A newly designed quick-hitch device for automatically changing the attachment will also be equipped. In the next step of dynamic robot model research, hydraulic servo control research will be carried out to change the attachment of the HULUWA demolition robot automatically.

## Figures and Tables

**Table 1:** Modified D-H parameters of demolition robots.

Joint i	*θ*_i_	d_i_	α_i_	a_i_
1	*θ_1_*	0	0	0.68 m
2	*θ_2_*	0	90°	0.515 m
3	*θ_3_*	0	0	0.82 m
4	*θ_4_*	0	0	1.415 m
5	*θ_5_*	0	0	0.938 m

**Table 2:** Data of joint {4} docking points.

Docking Point	Position	*RZ*_4_	Distance Error
Docking Point 1	(−0.864 m, 0, 0.709 m)	−70.9°	0.017 m
Docking Point 2	(−0.717 m, 0, 0.857 m)	−81.6°	0.064 m
Docking Point 3	(−0.557 m, 0, 0.969 m)	−91.6°	0.134 m
Docking Point 4	(−0.380 m, 0, 1.051 m)	−101.6°	0.237 m
Docking Point 5	(−0.192 m, 0, 1.101 m)	−111.6°	0.377 m

**Table 3:** Time consumption of attachment changing with trajectory planning and without trajectory planning.

Attachment Changing Stage	Without Trajectory Planning	Trajectory Planning
Initialization	150 s	60 s
Preparation	55 s	65 s
Range Alignment	30 s	5 s
Angle Alignment	15 s	5 s
Total Time	250 s	135 s

## Supplementary Materials

The following are available online at https://youtu.be/4m-wow-ABio: Video S1: Research on Trajectory Planning of Demolition Robot Attachment Changing; Video S2: Demolition Robot Attachment Changing without Trajectory Planning (https://youtu.be/vkGAPH_W734).
